# Using WhatsApp as a Quick-Access Personal Logbook for Maintaining Clinical Records and Follow-Up of Orthopedic Patients

**DOI:** 10.7759/cureus.12900

**Published:** 2021-01-25

**Authors:** Arvind Kumar, Yawar Haider, Mukesh Kumar, Rizwan Khan

**Affiliations:** 1 Orthopaedics, Hamdard Institute of Medical Sciences and Research, New Delhi, IND; 2 Orthopaedics, Jamia Hamdard, New Delhi, IND

**Keywords:** clinical records, data collection, logbook, research, whatsapp

## Abstract

Record keeping is an important aspect of orthopedic teaching, training, and practice for both documentation purpose and academic use. Physical logbooks may act as important documents but do not provide quick searchable access to the data they contain. Through this technique, we describe a simple method of creating personalized clinical records/logbook on WhatsApp through which individual records can be accessed with minimal basic key information in a span of a few seconds.

## Introduction

Record keeping is an important aspect of orthopedic teaching, training, and practice for both documentation purpose and academic use. While data can be safely stored in hard drives, in mobile storage devices, and even on cloud services, data segregation consumes a lot of time. Even if data are segregated in specific folders, searching for a particular diagnosis, time frame, and patient details is not easy and consumes precious time. Physical logbooks may act as important documents but do not provide quick searchable access to the data they contain. Suppose, for example, if you are interested in clinical pictures and radiographs of a complex distal femoral fracture you operated in May 2020 and this is the only information you have, then you would search through a bunch of images, folders, or database to find that particular case. This will cost a considerable amount of time. Any handy method that provides the required information in a few seconds would be desirable. Smartphones are handy and easily-accessible devices, but segregation of data on them is difficult. The WhatsApp messenger has revolutionized communication and health-care delivery [1]. Through this brief communication, we describe a simple method of creating personalized clinical records/logbook on WhatsApp through which individual records can be accessed with minimal basic key information in a span of a few seconds.

## Technical report

Step 1: creating customized WhatsApp groups

**Figure 1:**
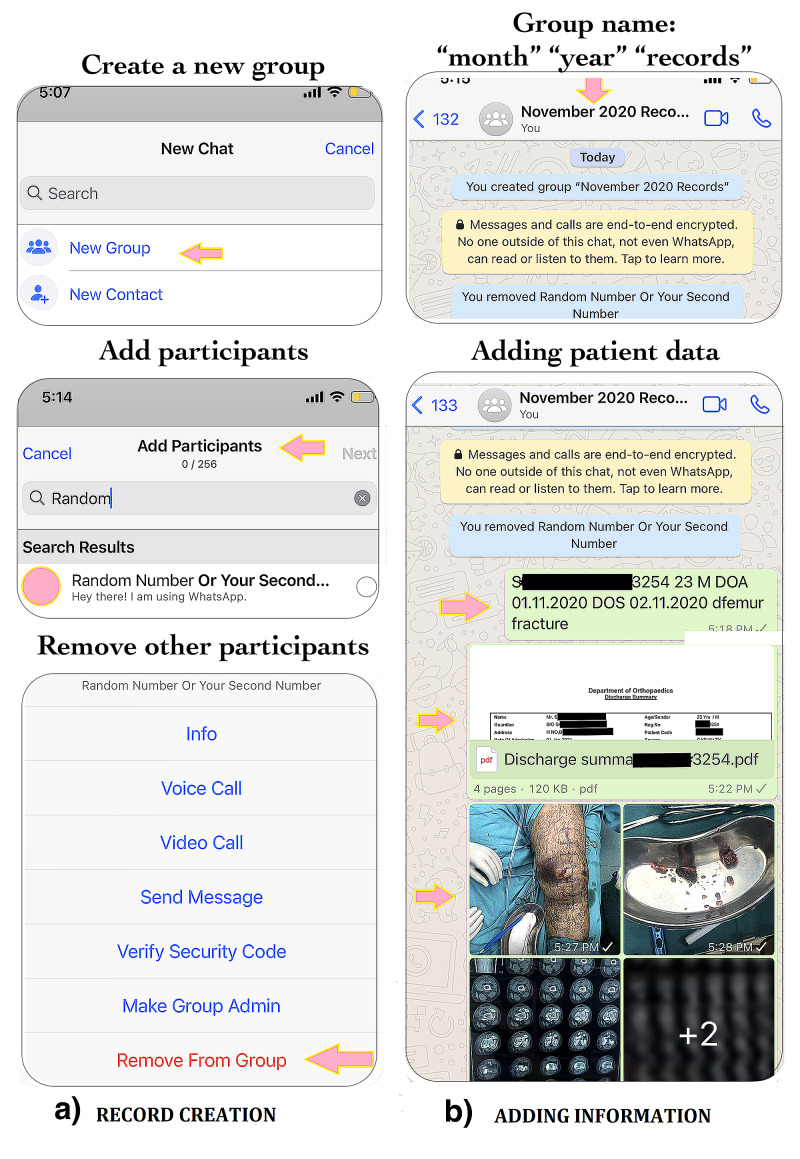
a) Record creation: creation of a WhatsApp group with you as the solo member. The second number that needs to be added can be your other number or a number which you can remove after group creation. b) Adding information: the group needs to be named as “month” + “year” + “records”. Patient details including name, registration info, date of admission, and surgery should be put as a message followed by discharge summary, clinical pictures, and other records in a continuous thread.

Using the "create group" link on WhatsApp, create a group that includes only your WhatsApp accounts (primary and secondary numbers) as the members (Figure 1a). Although it is desirable to have at least two WhatsApp accounts, you can create a solo member account as well by adding one of your colleagues/friends as the member of the group and removing his/her account after creating the group. Name the group as “month name” <space> “associated year” <space> "records”. By this method, you can create month-based groups like Jan 2019 records, Feb 2019 records, etc.

Step 2: adding data to the groups

Once a month-based group is created, you need to add the data of individual patients that were admitted or operated during that month (Figure 1b). For each patient, you need to add a message that contains the patient name, age, sex, registration number, date of admission, date of surgery, and a code suggesting the location of the orthopedic disorder and another code suggesting the nature of the orthopedic disorder. The latter two can be framed according to the readers’ preference. We, for instance, use the letter “p” for proximal, none for mid, and “d” for the distal part of the affected bone. For pathologies involving joint, joint names can be added. Therefore, if a patient had a neck femur or intertrochanteric fracture, “pfemur fracture” can act as the diagnostic code. However, the readers are free to use their own coding system, as our version is only for an illustration purpose. This is followed by the addition of a PDF file of the discharge summary of the concerned patient. The discharge PDF file can also be added to the WhatsApp group through a PC by selecting the WhatsApp desktop option on the mobile application. This is followed by adding the clinical pictures and radiographs and other investigations that are available. Additional information regarding the exact folder location on the hard drive or PC or the online link can also be added in the message thread. Subsequently, the data of other patients can be added in a similar manner. For cases with multiple location-based diagnoses, multiple diagnosis-based codings can be used. For example, if a patient sustains two fractures, one distal radius and another distal femur, then a code of "dradius" corresponding to the distal radius and "dfemur" corresponding to the distal femur can be added with fracture in the end. Searching for one diagnosis-based code would yield the multiple diagnosis-based results as well while searching for a combination of two or more diagnosis-based codes in the search box would screen the patients with combined injuries only.

Step 3: accessing the stored data with minimal key information

**Figure 2:**
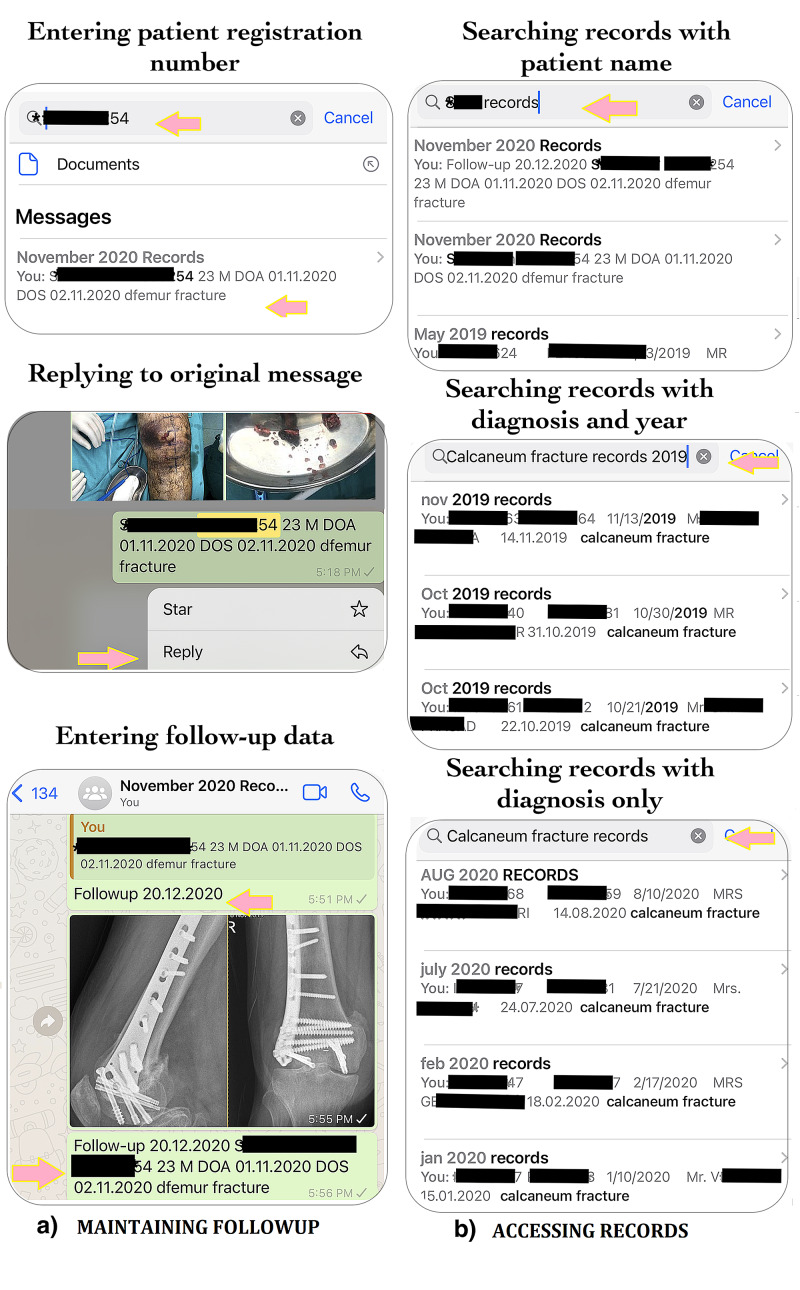
a) Maintaining follow-up: the patient information is searched through the WhatsApp search box using specific key information such as registration number. A reply is added to the original message with the follow-up date followed by the clinical information that needs to be added. At the end, the original first message is copy-pasted after writing the follow-up date. b) The records can be searched using several combinations of key information such as patient name, diagnosis, month, and year with the common term “records”. More key information will yield more precise results.

Figure 2 shows how to access the stored data with minimal key information. The registration numbers of patients are the most specific keywords for checking records during their follow-up visits (Figure 2b). But, occasionally, you remember only the diagnosis of the case when in need of certain clinical pictures, radiographs, or other records. In such a situation, the diagnosis-based code words along with “records” word unique to the groups created can be searched through the WhatsApp search box. The search results will yield a list of all cases with that diagnosis-based code in chronological order. Adding the patient name or month and year or just the year would help in more precise results. For example, if you need the clinical records of a patient of calcaneum you operated in 2019 but are not sure of the patient name, registration number, or any other key information, just search for the diagnosis-based code, a year, and with “records” word, in the end, i.e., calcaneum(location)<space>fracture (disorder)<space>2019(year)<space>records. The search results will yield a list of all cases of calcaneum fractures that you operated in 2019 and that were added to the records. Even if you do not remember the year, the search results will yield the diagnosis-based list in chronological order. Adding the month or name of the patient will yield more precise results.

Step 4: maintaining follow-up records

Another advantage of the described technique is the easy maintenance of follow-up records (Figure 2a). Search the registration number or appropriate key information as discussed above in WhatsApp. A reply can be added to the original post and the follow-up information can be added in that reply. The original thread can then be copy-pasted at the end with follow-up date to make easy identification of the follow-up data. Next time when you search for the patient records, you can easily differentiate from the original and the follow-up records as search results will display both.

## Discussion

The concept of the application of electronic devices in clinical data and information collection is not new [2]. There had been several articles concerning the maintenance of electronic logbooks (e-logbooks) on smartphones and personal computers [3-6]. Moreover, the use of smartphones is quite high among healthcare professionals [7]. Most of the e-logbooks create electronic records of patient data in mobile storage or cloud storage. A number of well-rated applications are available on Apple App Store and Google play store that can be satisfactorily used for entering the patient data and retrieving them when required. Surgical Logbook by Surgilog, Surgeon Logbook Pro, Surgery Notebook, Surgical Logbook, and Universal Logbook are among the top-rated smartphone-based logbook applications [4]. Some of these applications are free of cost while some are paid ones. However, free applications are equally effective compared to the paid ones. The paid applications offer additional advantages of unlimited cloud storage space for data collection and better backup services. The chief limitations that these applications pose are that they may not be easily operable by all clinicians. Most of these applications open up a list of dropdown selection menus for the purpose of entering maximum and relevant patient information that might not be relevant to or required by the clinician. For example, when searching for relevant records, the surgeon might not be interested in the date of birth of the patient, the exact starting point and ending point of the procedures performed, and more detailed technical information. Therefore, the acceptability criteria of the ease of use these applications will vary from clinician to clinician. There is a need for validation studies or surveys to establish an ideal logbook for orthopedic surgeons. Another drawback of these logbooks is the lack of an easy method for maintaining patient follow-up. No separate section or function has been provided for follow-up maintenance in these applications. Another concern is that the data entered may not be secure in all of the logbook applications. Some are password-protected while others are not. Our technique appears to address the majority of these concerns. Firstly, the clinician can choose the data he/she wants to carry as a record. We used patient name, age/sex, registration number, date of admission and surgery, diagnosis, and follow-up date. These are the basic data and can be simply typed on-the-go without tapping on the list of drop-down menus or can be simply copy-pasted from hospital records. Secondly, the clinicians might not be familiar with the usage of the logbook applications, and some may even find it difficult and more time-consuming to enter data on those. WhatsApp, on the other hand, has a large outreach and even non-medical personnel are familiar with its usage. Lastly, the data entered on WhatsApp has end-to-end encryption, which keeps it secure. Additionally, the currently described technique of surgical logbook maintenance has several other advantages, which include the following:

1. Maintenance of an “on-the-go” low-cost personal logbook.

2. As WhatsApp syncs data to your google and iCloud accounts, data remain backed up.

3. Helpful in quick access to all clinical records for a specific diagnosis, patient, and time frame. Figure 2 shows effective examples of each.

4. Potential tool for research work and paper writing with all information readily available.

5. Monthly records are helpful for teaching activities, clinical meetings, audits, and introspections.

6. The group chats can anytime be exported as a document file and can be attached to physical logbooks as well.

There are some limitations to the current technique of logbook maintenance. Firstly, even though most of the clinicians are familiar with WhatsApp usage, the same may not be applicable to the data entering steps described in this note. An initial period of trial and testing would be required for the application of this technique. Secondly, the figures and images on WhatsApp message threads might not be of optimum quality. The same can be addressed by uploading high-quality pictures, adding links from cloud storage, and attaching important images as files or documents rather than as images format. Lastly, the current technique describes the authors' and their colleagues' experience in this technical note, and the experiences of other clinicians may vary. Nevertheless, the current technique provides a potential alternative to smart logbook applications using a simple method and without the need for any additional free or paid applications other than WhatsApp.

## Conclusions

We feel that the described technique will be helpful for the clinicians especially the budding orthopedists who need to be well versed with the importance of record keeping. Initially, it may take some time to become familiar with the process, but with time this method of record-keeping will help a lot in several orthopedic domains.
